# Strategy to avoid local recurrence in patients with locally advanced rectal cancer

**DOI:** 10.1186/s13014-019-1253-9

**Published:** 2019-03-27

**Authors:** Takatoshi Nakamura, Takeo Sato, Kazushige Hayakawa, Wasaburou Koizumi, Yuji Kumagai, Masahiko Watanabe

**Affiliations:** 1 Department of Surgery, Kitasato University School of Medicine, 1-15-1 Kitasato, Minami-ku, Sagamihara, Kanagawa Japan; 2 Department of Radiology and Radiation Oncology, Kitasato University School of Medicine, 1-15-1 Kitasato, Minami-ku, Sagamihara, Kanagawa Japan; 3 Department of Gastoroenterology, Kitasato University School of Medicine, 1-15-1 Kitasato, Minami-ku, Sagamihara, Kanagawa Japan; 4 Director of Clinical Trial Center, Kitasato University School of Medicine, 1-15-1 Kitasato, Minami-ku, Sagamihara, Kanagawa Japan

**Keywords:** Neoadjuvant Chemoradiotherapy, Rectal Cancer, New treatment strategy

## Abstract

**Background:**

To clarify the short- and long-term outcomes of radical surgery after neoadjuvant chemoradiotherapy (NCRT) with TS-1 and irinotecan, which enhances radiosensitivity, in patients with locally advanced rectal cancer.

**Methods:**

The study group comprised 105 patients with locally advanced rectal cancer who received NCRT followed by radical surgery. NCRT consisted of pelvic radiotherapy (45 Gy in 25 fractions over a period of 5 weeks), S-1 (80 mg/m^2^) given concurrently for 25 days, and irinotecan (60 mg/m^2^), given once a week as a continuous intravenous infusion. Radical surgery was performed 8 weeks after treatment.

**Results:**

A pathological complete response was confirmed in 23.8%. The 5-year recurrence-free survival rate was 79.3%, and the 5-year overall survival rate was 87.1%. Multivariate analysis showed that the following 4 variables were independent predictors of recurrence-free survival: Sex (male: *p =* 0.0172), Pre-treatment tumor diameter (< 40 mm: *p =* 0.0223), Histopathological treatment response (grade 0,1: *p =* 0.0169), and ypN (ypN1: *p =* 0.1995; ypN2: *p =* 0.0007). Only ypN was an independent predictor of overall survival (ypN1: *p =* 0.0009; ypN2: *p =* 0.0012).

**Conclusions:**

Our treatment strategy combining TS-1 with irinotecan to increase radiosensitivity had a high response rate.

## Background

Improved outcomes and the control of local recurrence have long been important goals in the treatment of locally advanced lower rectal cancer. Preoperative radiotherapy has been demonstrated to be associated with better compliance and a lower incidence of complications than postoperative radiotherapy [1]. Many prospective clinical studies have shown that preoperative radiotherapy combined with chemotherapy can significantly decrease the rate of local recurrence [2]. At present, total mesorectal excision (TME) combined with preoperative chemoradiotherapy has become the main standard treatment in Western countries [3]. However, whether preoperative radiotherapy should be given according to a short-course or long-course schedule remains controversial. In addition, to prevent defecatory disturbances and late complications caused by radiotherapy, clinical trials have studied whether neoadjuvant chemotherapy can be given instead of radiotherapy [4].

In Japan, a retrospective study demonstrated that TME plus lateral lymph-node dissection (LLD) can decrease the rate of local recurrence in patients with lower rectal cancer. This procedure has therefore become standard treatment. A randomized, controlled trial was performed to determine whether TME alone is noninferior to TME plus LLD as standard treatment [5]. The results showed that TME plus LLD was more invasive and associated with a higher incidence of complications than TME alone. However, the noninferiority of TME alone was not demonstrated on long-term follow-up, and the local recurrence rate was significantly lower in the TME plus LLD group. TME plus LLD has thus retained its position as standard treatment [6]. In our study, TME was conducted without performing LLD in patients whose lateral lymph nodes were not swollen before surgery because they had received radiotherapy up to the lateral region at the time of NCRT. In the TME study, preoperative treatment was standard therapy and was showed to be fully effective. In patients with swollen lateral lymph nodes before surgery, LLD should be performed in addition to preoperative therapy to achieve local control.

Prophylactic LLD is considered adequately effective in patients who receive NCRT. In patients with swollen lateral lymph nodes before surgery, we confirmed the treatment response, carefully considered the advantages and disadvantages of LLD, and performed LLD to achieve local control.

S-1 is a combined preparation of tegafur, oteracil, and gimeracil. Gimeracil can enhance radiosensitivity. Irinotecan enhances the effectiveness of 5-fluorouracil converted from tegafur. To further decrease local recurrence and increase the survival rate, we previously designed a regimen that combined LLD with neoadjuvant chemoradiotherapy (NCRT) including S-1, irinotecan, and radiotherapy to decrease the local recurrence rate and increase the survival rate. We conducted Phase 1 and 2 studies and showed that this regimen was safe and effective, with a very low rate of local recurrence [7–9]. However, LLD was associated with an increased bleeding volume, adverse effects on urination and sexual function, and increased difficulty in laparoscopic surgery. To minimize surgical damage and prevent postoperative complications, we designed a new regimen for NCRT. LLD was omitted, and the irradiated region was extended laterally. Therapeutic LLD was performed only in patients with enlarged lateral lymph nodes on preoperative imaging studies to eradicate local recurrence by dissection and chemoradiotherapy.

The aim of our study was to evaluate the effectiveness and the safety of this new regimen for NCRT and to clarify the long-term outcomes, particularly with respect to whether the risk of local recurrence was decreased.

## Methods

### Subjects

**Fig. 1:**
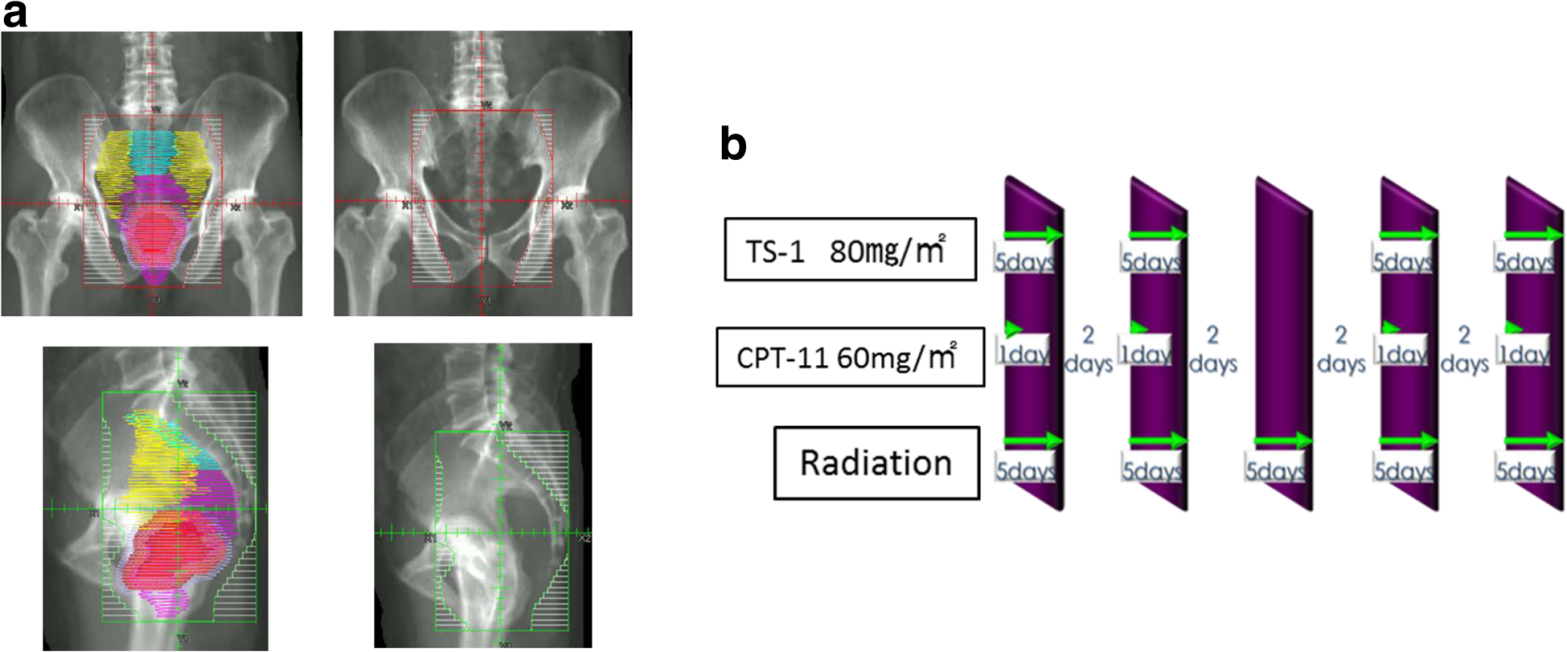
As for the clinical target volume (CTV), the superior border did not go beyond the L5-S1 interspace, the lateral border did not go beyond the outer edge of the lesser pelvic cavity, and the posterior border did not go beyond the pelvic surface of the sacrum (**a**). TS-1 (80 mg/m2) was given orally after breakfast and dinner on days 1 to 5, days 8 to 12, days 22 to 26, and days 29 to 33. Irinotecan (60 mg/m2/day) was given as a continuous intravenous infusion over a 90-min period on days 1, 8, 22, and 29 (**b**)

The study group comprised 105 patients with advanced lower rectal cancer who were treated in our hospital from January 2011 through December 2015 (Fig. 1). Risk factors for recurrence, 5-year disease-free survival (DFS) rates, and 5-year overall survival (OS) rates were studied. Our protocol was approved by the institutional ethics committee of Kitasato University Hospital (Kanagawa, Japan) on June 19, 2017 (B17–063). Written informed consent was obtained from all patients. Eligible patients had to have a histopathologically confirmed diagnosis of previously untreated rectal adenocarcinoma and an Eastern Cooperative Oncology Group performance status of 0 to 3. Pathologic staging was determined according to the 7Th Edition of the TNM Staging System (Union for International Cancer Control). Other inclusion criteria were an age of 20 to 82 years at the time of enrollment and no severe dysfunction of major organs (including the bone marrow, heart, lung, liver, and kidneys). The preoperative diagnosis was based of the results of barium enema examination, colonoscopic examination including histopathological examinations, computed tomography (CT), and magnetic resonance imaging (MRI), and the absence of distant metastasis was confirmed. The height of tumors was not included in the inclusion criteria. Lymph-node metastasis was defined as lymph nodes with a short-axis diameter of 7 mm or greater on MRI. The site of the tumor was determined by measuring the distance between the lower edge of the tumor and the anal verge before treatment. The pros and cons of anal sphincter preservation were evaluated on the basis of tumor location.

### Radiotherapy and chemotherapy

Radiotherapy was administered in a dose of 1.8 Gy once daily 5 days per week. A total of 25 fractions were administered (total dose, 45 Gy), using a three-field (bilateral and posterior) technique or a four-field (anteroposterior and bilateral) technique. All 3 or 4 fields were irradiated at each session of radiotherapy. Lymph nodes included the mesorectum (pararectal lymph nodes), internal iliac lymph nodes, and obturator lymph nodes. As for the clinical target volume (CTV), the superior border did not go beyond the L5-S1 interspace, the lateral border did not go beyond the outer edge of the lesser pelvic cavity, and the posterior border did not go beyond the pelvic surface of the sacrum (Fig. 1a). TS-1 (80 mg/m^2^) was given orally after breakfast and dinner on days 1 to 5, days 8 to 12, days 22 to 26, and days 29 to 33. Irinotecan (60 mg/m^2^/day) was given as a continuous intravenous infusion over a 90-min period on days 1, 8, 22, and 29 (Fig. 1b). The histologic response rate of the primary tumor was evaluated according to the grade of the response. However, the histologic response rate of lymph nodes was not evaluated.

### Treatment schedule and criteria for changes in treatment regimens

In our protocol, treatment was temporally discontinued if grade 3 or higher diarrhea and vomiting developed. Blood and urine tests were performed every week to investigate hematologic toxicity and renal toxicity. The dose was reduced or treatment was postponed on the basis of the results. Toxicity was evaluated according to the second edition of the Common Terminology Criteria for Adverse Events (CTCAE), issued by the National Cancer Institute. If toxicity requiring dose reduction occurred within a course of treatment, the dose of irinotecan was decreased by 1 level (20%), and treatment could be resumed. If toxicity requiring dose reduction occurred after the dose had been decreased by 1 level, chemotherapy was discontinued without further dose reduction.

### Surgery

Open surgery was performed during the first half of the study, and laparoscopic surgery was performed during the second half. In both groups, the autonomic nerves were preserved bilaterally, and TME was performed. Prophylactic lateral lymph-node dissection was not done. In patients who had enlarged lateral lymph nodes with a short-axis diameter of 7 mm or greater on imaging studies before treatment, the ipsilateral middle rectal, internal iliac, and obturator lymph nodes (lateral lymph nodes) were dissected. The distal portion of the rectum was incised, while securing a distance of at least 2 cm from the lower edge of the tumor. If the distance could not be secured, abdominoperineal resection was performed. A temporary ileostomy was performed in all patients. The surgical wound was closed after confirming the absence of suture failure and anastomotic stricture 3 to 6 months after surgery.

### Evaluation of pathologic specimens

The antitumor effectiveness of NCRT was evaluated histopathologically using serial sections of the resected specimen obtained at 5-mm intervals. The degrees of cancer cell degeneration, necrosis, and fusion were assessed. The evaluation criteria were based on the histopathological classification proposed by Dworak et al. [10] and the Japanese Classification of Colorectal Carcinoma [11]. Grade 1a and grade 1b were similarly classified as Grade 1. The histopathological response of the tumor was evaluated according to the Tumor Regression Grade (TRG) as follows: Grade 0 (no response), no discernible treatment-induced degeneration or necrosis of the cancer cells; Grade 1 (mild response), degeneration, necrosis, or fusion of less than about two-thirds of the cancer cells; Grade 2 (substantial response), marked degeneration, fusion, or disappearance of at least two-thirds of the cancer cells; Grade 3 (complete response), necrosis of all cancer cells or fusion or disappearance of all cancer cells and replacement by granuloma-like or fibrous tissue.

### Follow-up survey

During preoperative chemotherapy and radiotherapy, medical examinations were performed in the Department of Radiology on the days of radiotherapy. Blood samples were obtained before treatment, and irinotecan was administered once per week a total of 4 times. Blood samples were obtained in the outpatient clinic every 2 to 3 weeks after the completion of preoperative treatment. The following variables were assessed in the outpatient clinic: medical history; the results of physical examinations; performance status; carcinoembryonic antigen (CEA) and cancer antigen (CA) 19–9 levels; blood cell counts and serum chemistry; the results of CT of the chest, abdomen, and pelvis; the results of barium enema examinations; and the results of colonoscopic examinations. After the completion of 5 courses of NCRT, barium enema examination, CT of the chest, the abdomen, and the pelvis, rectal MRI, and biopsy of colonoscopic specimens were performed within 8 weeks before surgery to evaluate clinical response. For follow-up after surgery, the patients were evaluated every 3 months during the first 2 years, every 6 months during years 3 to 5, and every 12 months subsequently. Recurrence was diagnosed on the basis of the results of CT, positron emission tomography, MRI, and colonoscopic examinations, including the results of biopsy and cytologic examinations if possible.

### Postoperative chemotherapy

Patients with ypN1 or ypN2 disease were given 6 courses of adjuvant chemotherapy with FOLFOX.

### Statistical analysis

Descriptive statistics and distributions were calculated for demographic variables. The effects of demographic variables on the 5-year DFS and 5-year OS were investigated as long-term outcomes. DFS was defined as the interval from the date of starting treatment to the date of recurrence. OS was defined as the interval from the date of enrollment to the date of death from any cause. The following 12 demographic variables were studied: sex, age, tumor location, clinical tumor stage, tumor diameter before treatment, CEA level at initial examination, CA19–9 level, whether or not NCRT was completed, surgical procedures, TRG, ypN, and ypCR.

The 5-year DFS and 5-year OS were calculated according to each demographic variable by the Kaplan-Meier method. A log-rank test was used to evaluate the influence of each variable. Variables with *P* values of less than 0.1 in a log-rank test were designated as candidate explanatory variables. A Cox proportional-hazards model was used to select the variables (stepwise forward selection method: *P* values of < 0.1 as calculated by the Wald test were regarded as standard). The hazard ratio and the 95% confidence interval of each explanatory variable in the final model were calculated. Statistical analysis was performed with the use of SPSS version 8, OJ (SPSS, Chicago, USA).

## Results

### Toxicity of NCRT

The demographic characteristics of the patients in our study are shown in Table 1. The completion rate of our regimen was 85.7% (90 patients). Treatment was discontinued in 5 patients, and dose reduction was performed in 10 patients. No patient died of NCRT-related causes. Grade 3 adverse events occurred in 11 patients (10%): 6 patients (6%) had diarrhea, and 5 patients (5%) had neutropenia.

**Table 1:**
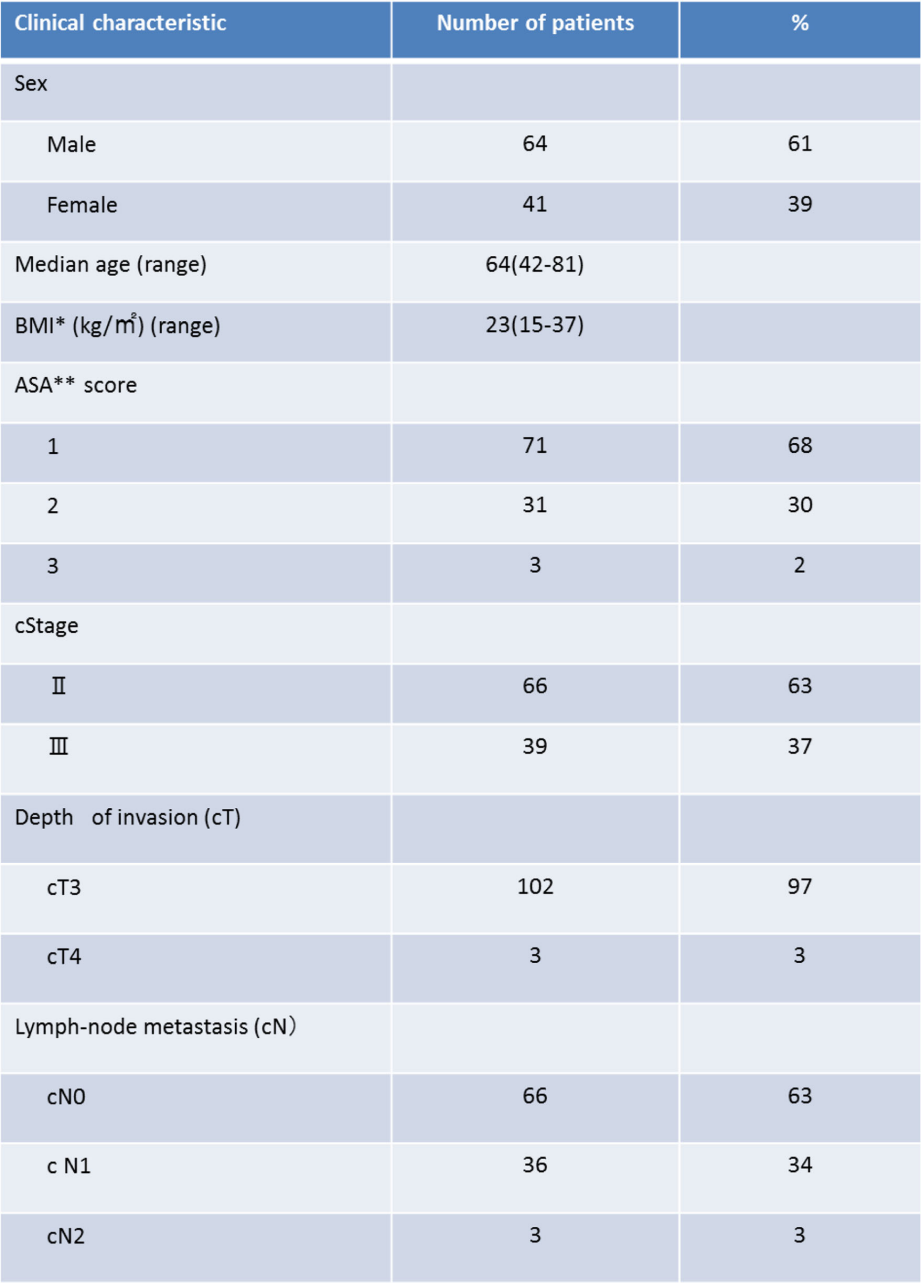
Clinical characteristics of 105 patients with rectal cancer

BMI*; Body mass index, ASA**;American society anesthesiologists

### Short-term outcomes

Open surgery was performed in 37 patients (35.2%) during the first half of the study period. Laparoscopic surgery was performed in 68 patients (64.8%) during the second half of the study period: low anterior resection was done in 55 patients (52.4%), abdominoperineal resection in 48 patients, internal anal sphincter resection in 1 patient, and Hartmann’s procedure in 1 patient. Postoperative complications occurred in 29 patients (27.6%): suture failure occurred in 7 patients (7/55, 12.8%), small bowel obstruction in 9 patients (9/105, 8.6%), perineal wound infection in 5 patients (5/48, 10.4%), transient dysuria in 2 patients (2/105, 1.9%), enteritis in 2 patients (2/105, 1.9%), midline wound infection in 1 patient, pelvic abscess in 1 patient, chylous ascites in 1 patient, aspiration pneumonia in 1 patient, and pulmonary infarction in 1 patient. No patient underwent reoperation after surgery. The median length of the postoperative hospital stay was 14 days (range, 8 to 62). There were no surgery-related deaths.

### Evaluation of histopathological response (TRG)

The number of examined lymph nodes (mean ± SD) was 12 ± 8.8. Overall, 21 patients (20.0%) had ypN1 disease, and 7 patients (6.7%) had ypN2 disease. LLD was performed in 14 patients (14/105, 13.3%). Two of these patients (2/105, 1.9%) had metastasis. Lateral lymph-node metastasis was found in 2 patients: 1 patient had metastasis to the obturator lymph nodes in and the other had metastasis to the internal iliac lymph nodes. In 25 patients (25/105, 23.8%), pathological examination showed no remnant cancer cells (ypCR). The TRG was Grade 3 in 27 patients (27/105, 25.7%). However, 2 of these patients (2/27, 7.4%) had lymph-node metastasis despite a response of TRG 3. TRG 2 was found in 23 patients (23/105, 21.9%), and 7 (7/23, 30.4%) of these patients had lymph-node metastasis. TRG 1 was found in 49 patients (40.6%), and 17 (17/49, 34.7%) of these patients had lymph-node metastasis. TRG 0 was found in 6 patients (5.7%), and 2 (2/6, 33.3%) of these patients had lymph-node metastasis.

### Prognostic factors

The median follow-up period was 52 months (range, 16 to 88). The 5-year DFS rate was 79.3%, and the 5-year OS rate was 87.1%. Recurrence occurred in 20 patients (19.0%). The site of initial recurrence was the lung in 8 patients, the liver in 7 patients, the para-aortic lymph node region in 4 patients, and bone metastasis in 1 patient. No patient had local recurrence.

**Fig. 2:**
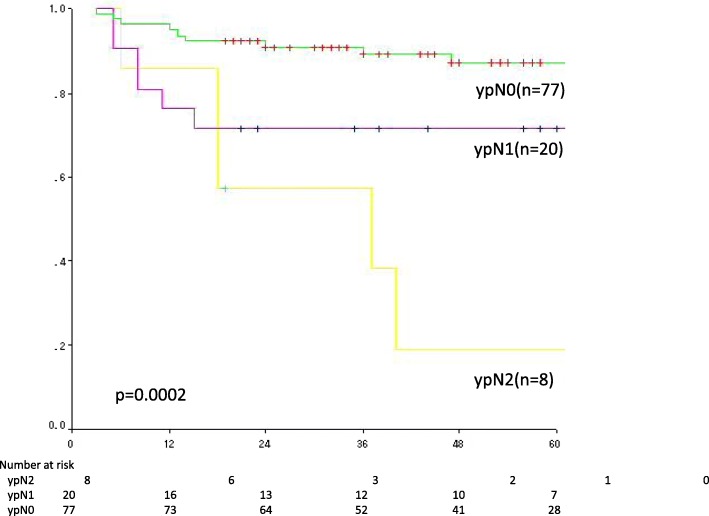
Candidate prognostic factors were selected by multivariate analysis (Cox proportional-hazards model) (Wald test, *p* < 0.1). Candidate prognostic factors for DFS were sex (**a**), tumor diameter before treatment (≥40 mm vs. ≤39 mm) (**b**), clinical tumor stage, TRG (Grade 0 or 1 vs. Grade 2 or 3) (**c**), ypN (1 or 0 vs. 2 or 0) (**d**), and ypCR. Candidate prognostic factors for OS were sex, tumor diameter before treatment, and ypN (**e**)

**Fig. 3:**
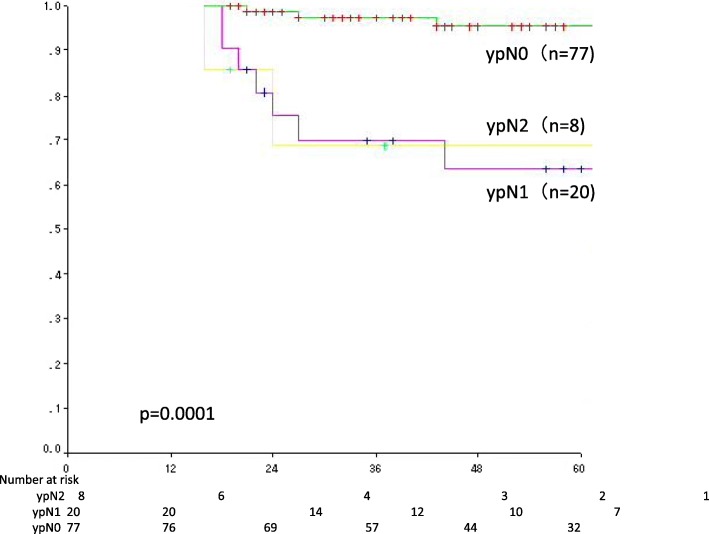
The results of multivariate analysis showed that sex, tumor diameter before treatment, TRG, and ypN were prognostic factors for DFS, whereas only ypN was a prognostic factor for OS

Univariate analysis was performed to evaluate the influence of each prognostic factor on DFS and OS and to identify candidate prognostic factors (log-rank test, *p* < 0.1). Candidate prognostic factors were selected by multivariate analysis (Cox proportional-hazards model) (Wald test, *p* < 0.1). Candidate prognostic factors for DFS were sex, tumor diameter before treatment (≥40 mm vs. < 40 mm), clinical tumor stage, TRG (Grade 0 or 1 vs. Grade 2 or 3) (Fig. 2c), ypN (1 or 0 vs. 2 or 0) (Fig. 2), and ypCR. Candidate prognostic factors for OS were sex, tumor diameter before treatment, and ypN (Fig. 3, Table 2). The results of multivariate analysis showed that sex, tumor diameter before treatment, TRG, and ypN were prognostic factors for DFS, whereas only ypN was a prognostic factor for OS (Table 3).

**Table 2:**
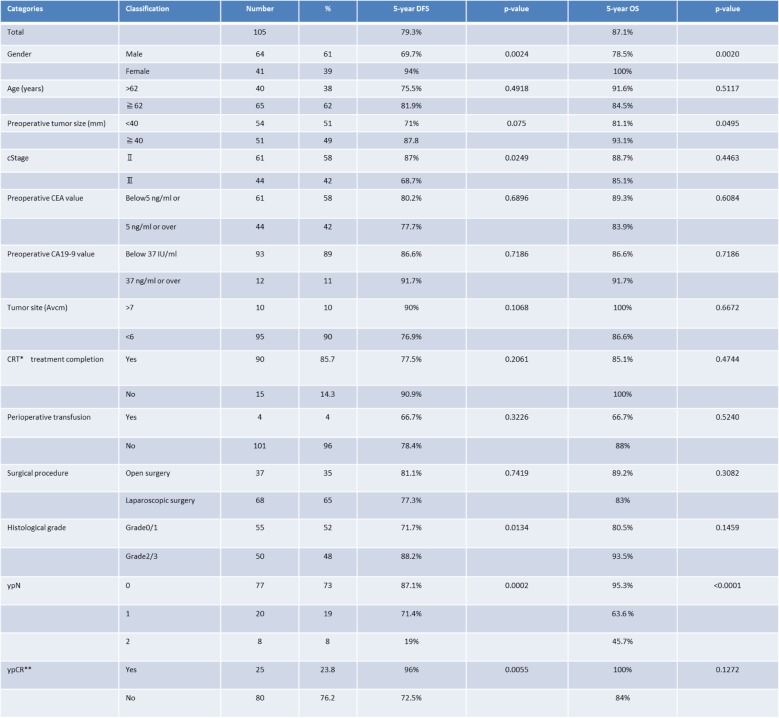
Univariate prognostic analysis in 105 patients with rectal cancer who underwent NCRT

CRT*;chemoradiotherapy, CR**;complete response

**Table 3:**
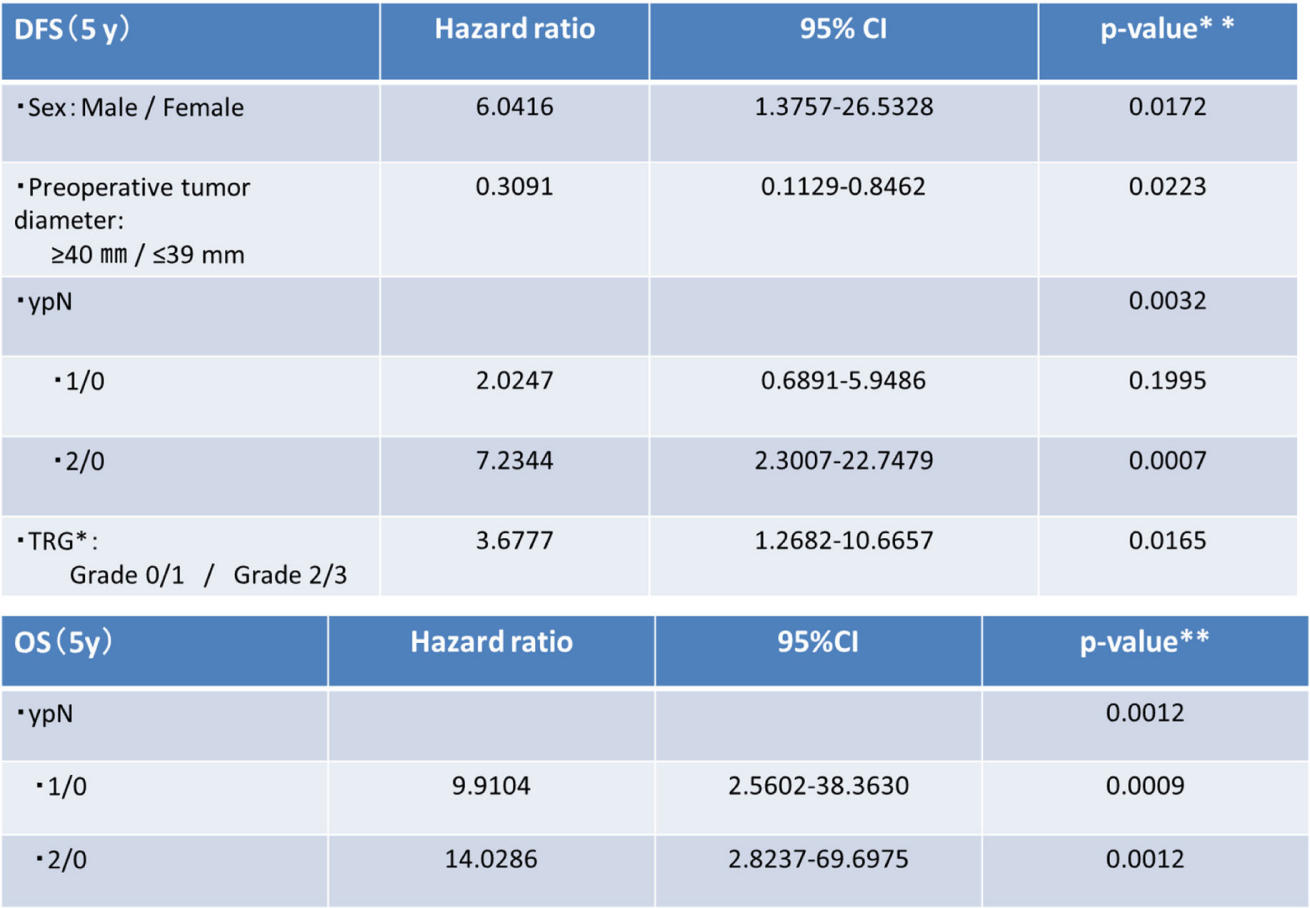
Multivariate prognostic analysis for DFS and OS

TRG*;Tumor Regression Grade, **Cox proportional-hazards model (Wald test)

## Discussion

In the present study, we examined prognostic factors and the effectiveness of a new regimen consisting of NCRT with S-1 plus irinotecan, TME, and therapeutic LLD in patients with locally advanced rectal cancer and found that NCRT was associated with a low incidence of grade 3 or 4 adverse events. Treatment could be safely performed and had a high completion rate. The ypCR response rate was relatively high (23.8%). It is noteworthy that no patient had local recurrence. The major route of initial recurrence was hematogenous spread to the lung and liver metastasis. The 5-year DFS rate was 79.3%, and the 5-year OS rate was 87.1%, indicating relatively good long-term outcomes. Our regimen was safe with a high response rate and completely inhibited local recurrence, achieving good outcomes. Sex, tumor diameter, TRG, and ypN(+) were identified as prognostic factors, and the long-term outcomes of patients with ypN2 disease were extremely poor.

The safety of CRT depends on factors such as patients’ general condition, the extent and dose of radiotherapy, and the type and dose of chemotherapy. It is generally known that as compared with surgery alone, the incidence of adverse events is increased by neoadjuvant radiotherapy (NRT) and is further increased by adding chemotherapy, leading to decreased treatment completion rates [2, 3, 12–14]. Given that FOLFOX is standard adjuvant chemotherapy for colon cancer, many studies have combined 5-fluorouracil-based chemotherapy with oxaliplatin to enhance the effectiveness of NCRT for rectal cancer. However, preoperative FOLFOX combined with radiotherapy was associated with a significantly increased incidence of adverse events including death and a decreased treatment completion rate in patients with rectal cancer [15–18]. Therefore, preoperative FOLFOX combined with radiotherapy must be carefully followed up in further studies and cannot be recommended at present. However, FOLFOX monotherapy is expected to be effective for rectal cancer similar to colon cancer and can therefore be used for preoperative chemotherapy and chemoselection. On the other hand, the completion rate of our NCRT regimen was high (85.7%) and the incidence of grade 3 or higher adverse events was only 10%, and no patient died. Radiotherapy combined with 5-fluorouracil and irinotecan was therefore considered safe and effective. However, this chemotherapeutic regimen combined with expanded-field radiation was associated with an increased incidence of adverse events. Therefore, careful setting of the radiation fields is essential for this regimen. S-1 is a combined preparation of tegafur, oteracil, and gimeracil. Gimeracil can enhance radiosensitivity. Irinotecan enhances the effectiveness of 5-fluorouracil converted from tegafur. To further decrease local recurrence and increase the survival rate, we previously designed a regimen that combined LLD with neoadjuvant chemoradiotherapy (NCRT) including S-1, irinotecan, and radiotherapy to decrease the local recurrence rate and increase the survival rate. Our study was characterized by the fact that hematological toxicity was most common. In other studies, hematological toxicity was less common, and many patients had dermal toxicity. There was no local recurrence, although many patients had hematogenous metastasis. Future measures may be needed. However, the DFS and OS rates were good [2, 3]. Our TNT protocol was associated with a higher treatment completion rate and a lower incidence of toxicity than other than the rates in other large controlled studies.

Because the local recurrence rate was decreased by combining chemotherapy with preoperative radiotherapy as compared with radiotherapy alone, the use of 5-fluorouracil-based NCRT has been recommended [1, 2, 5]. However, even when NCRT was combined with various drugs, including oxaliplatin, irinotecan, and molecular-targeted agents, the local recurrence rate could not be reduced to 0% [16, 17]. We combined NCRT with TME and additionally performed therapeutic LLD when lymph-node metastasis was diagnosed on MRI. LLD was performed in 14 patients who had enlarged lateral lymph nodes with a diameter of at least 7 mm on MRI. We believe that this led to the 0% rate of local recurrence, including lateral lymph-node recurrence. Akiyoshi at al [18]. also performed LLD in patients suspected to have lateral lymph-node metastasis on diagnostic imaging studies and reported that the 3-year DFS rate was good even among patients who were positive for lateral lymph-node metastasis. This was an epoch-making study because the presence or absence of lateral lymph-node metastasis, a conventional prognostic factor, did not become a prognostic factor in patients who received NCRT. Akiyoshi at al. reported that lateral lymph-node metastasis was found at the time of lateral lymph-node dissection in 40% of lateral lymph nodes that had a longest diameter of 7 mm or greater [18]. On the basis of this report, lateral lymph-node dissection was indicated for swollen lateral lymph nodes that had a diameter of 7 mm or greater on MRT before treatment. This description was added to the text. Our results and those of Akiyoshi at al. showed that NCRT combined with therapeutic LLD can inhibit local recurrence. On the other hand, our study showed that ypN2 disease was a very strong predictor of hematogenous metastasis and suggested that LLD can inhibit local recurrence and may facilitate selection of the optimal postoperative treatment regimen. We believe that further clinical studies are warranted to prospectively confirm the effectiveness of the strategy of combining therapeutic LLD with a preoperative treatment such as NCRT.

In general, the ypCR rate is 17 to 19.2%. In our study, however, the rate of ypCR in patients who received NCRT was relatively good (23.8%) [13, 19]. Given that the ypCR rate is higher after CRT than after preoperative radiotherapy, studies have been conducted to examine the rate of ypCR after combining 5-fluorouracil with oxaliplatin, irinotecan, and molecular-targeted agents. Because patients with ypCR had good outcomes, a new watch-and-see approach was recently been attempted. Patients who had a CR after CRT were divided into 2 groups: a watch-and-see group and a TME group. The local recurrence rate, DFS rate, and OS rate were compared between the groups, and one study reported that the differences were not significant [20]. Local resection of lesions that shrank after CRT or chemotherapy was attempted. To avoid curative surgery, which negatively affects patients’ quality of life, it is essential to perform effective multidisciplinary treatment, accurately diagnose pathological CR, and administer reliable salvage therapy. At present, however, diagnostic imaging studies are not practical for predicting ypCR because the sensitivity and specificity of fluorodeoxyglucose positron emission tomography is low [21].

Multivariate analysis was performed to examine prognostic factors and showed that DFS significantly correlated with sex, tumor diameter, ypN(+), and a TRG of 0 or 1. OS correlated with only ypN(+). Fokas et al. reported that ypN(+) and TRG significantly correlated with DFS and that ypN(+) and lymphatic invasion were significantly related to local recurrence on the basis of the results of CAO/ARO/AIO-94 trial [12]. Tumor diameter, ypN(+), and TRG thus represent treatment resistance, similar to tumor proliferation. As for treatment resistance, specific treatment regimens should be developed on the basis of the detailed genetic characteristics of tumor cells. In other words, new developments in precision medicine may play a key role in improving outcomes. In general, males have higher rates of complications, particularly suture failure, than females. Suture failure has long been know to be a prognostic factor for rectal cancer. Therefore, the development of more precise surgical techniques might contribute to an improvement in the outcomes of surgery [22, 23].

Our study demonstrated that a new NCRT regimen combining S-1 with irinotecan, which enhances the radiosensitivity of locally advanced rectal cancer, was a safe preoperative treatment with a high completion rate. Our results also showed that TME combined with therapeutic LLD can completely suppress local recurrence and improve DFS and OS. Further studies of biomarkers that can be used to predict ypCR are needed to improve outcomes, and new treatment regimens should be developed for treatment-resistant patients.

## Conclusions

Our treatment strategy combining TS-1 with irinotecan to increase radiosensitivity had a high response rate.
